# Proton radiobiology and its clinical implications

**DOI:** 10.3332/ecancer.2017.777

**Published:** 2017-10-26

**Authors:** Bleddyn Jones

**Affiliations:** Gray Laboratory, CRUK/MRC Oxford Oncology Institute, The University of Oxford, ORCRB – Roosevelt Drive, Oxford OX3 7DQ, UK

**Keywords:** proton therapy, radiobiology, RBE, linear energy transfer, cancer, ICRU

## Abstract

Denser ionisation clustering and complex DNA damage in proton Bragg peaks far exceeds that seen with conventional X-rays. This results in more efficient cell sterilisation, quantified by the relative biological effectiveness (RBE). Currently, a 1.1 RBE is used to determine the clinical proton doses by dividing the usual X-rays dose by this amount. This number, derived from short-term experiments, has been criticised as being irrelevant to late normal tissue (NT) effects following radiotherapy and included many control irradiations using lower voltage X-rays (with elevated RBE values) than those used in the clinic. In principle, an increased RBE could be used for each organ at risk, by using extensions of the clinically successful linear quadratic model.

Protons undoubtedly reduce or eliminate NT radiation dose in tissues distantly located from a tumour, but the necessity to include NT margins around a tumour can result in a higher volume of NT than tumour being irradiated. Deleterious side-effects can follow if the NT RBE exceeds 1.1, including in tissue very close to these margins and which are only partially spared.

Use of a constant 1.1 RBE can ‘overdose’ NT, which may require a greater dose reduction such as 1.2 in the brain; some tumours may be ‘under-dosed’ (since they might require a lesser or no reduction in dose). More sophisticated proton experiments show that RBE values of 1.1–1.5 and higher occur in some situations. There are now mathematical models of varying degrees of complexity that can estimate the RBE from the dose, LET and the low-LET radiosensitivities. True multidisciplinary cooperation is required to implement such new ideas in proton therapy in order to improve safety and effectiveness.

## Introduction

All therapeutic interventions can have adverse side effects as well as benefits, and proton therapy is no exception to this rule. Before any novel drug can reach its full potential, considerable background pre-clinical work is necessary, as well as standard trial-based forms of implementation and refinement in the clinic. In contrast, it is unfortunate that current legislation does not demand extensive pre-clinical work with radiation modalities.

Proton therapy remains the most promising form of radiotherapy because it can deliver ‘cancericidal’ doses while limiting the total energy deposition in the body, often avoiding unnecessary direct radiation doses to many organs in the body. In this way, protons reduce the volume of irradiated tissue as a consequence of the Bragg peak effect [1]. It follows that there is an expected reduction in some of the normal tissue morbidity and cancer induction experienced with conventional X-ray (or photon) beams because of their unnecessary traversion through all tissues in the beam direction. This review will not show physical dose maps or other Bragg peak features, since these are well illustrated elsewhere [1].

This ‘dose sparing’ advantage due to the Bragg peak is accompanied by two potential disadvantages that have to be overcome:

1. The placement of the Bragg peaks may be inaccurate due to physical reasons such as heterogenous tissue densities, patient movement and daily positioning or beam delivery related factors. Charged particle tracks also separate with increasing depth, due to Coulombic scattering caused by repulsive forces due to the positive charge of each particle). These issues are being addressed and are expected to become less significant with time due to improvements in dose computation and other technical aspects.
2. Of greater concern is the fact that within the Bragg peaks the pattern of energy deposition is in the form of closer ionisation events [2]. This causes increasingly clustered DNA damage which is more difficult or impossible for enzymatic cellular DNA repair mechanisms to restore, resulting in enhanced biological effects that can be equivalent to dose modification of between 1 and 3 in some situations. This could turn out to be advantageous within a cancer, but possibly deleterious for normal tissues close to the cancer.

Currently, the medical prescription of proton therapy dose includes a 10% dose reduction due to the expected enhanced bioeffectiveness. This figure is currently under dispute, and the present article summarises the clinical and scientific facts that underpin the normal tissue difficulties associated with enhanced bioeffectiveness. To understand this more fully, especially for the more general reader, it is necessary to describe:

1. Some essential aspects of radiotherapy, especially the inclusion of sometimes large margins of normal tissue beyond a cancer in order to eradicate the cells within its spreading edge which is not as well delineated, by imaging, as the cancer itself.
2. The physics and biology terms associated with enhanced bioeffectiveness, and how this is modified by dose and tissue characteristics.
3. The previous but influential research which suggested that the bioeffectiveness issues can be adequately compensated for by reducing the dose by 10% compared to megavoltage photons (X-rays) and why this must be incorrect.
4. What could be achieved with better modelling estimates for improved clinical decision making.

Also, some conclusions are made as to how to improve proton therapy, to make it safer and more effective within clinical trials or studies.

## Some essential physics and radiobiology

Along a particle or photon track the closeness of ionisations is assessed by the linear energy transfer (or LET), which essentially averages the energy released per micrometre distance of a proton particle track, independently of absorbed dose in a wider volume (which is the energy absorbed per kilogram of mass and expressed in units of Gy, in recognition of the British Physicist LH Gray).

The other essential definition is that of relative biological effect (or RBE). This essentially describes the dose modification required to maintain the same biological effect when LET is increased compared with a lower LET from another ‘control’ radiation. It is formally defined as a ratio, and in the context of clinical proton therapy is:

$$\mathrm{RBE}=\frac{\mathrm{Dose}\;\mathrm{of}\;\mathrm{the}\;\mathrm{control}\;\mathrm{megavoltage}\;\mathrm{photon}\;\mathrm{radiation}}{\mathrm{Dose}\;\mathrm{of}\;\mathrm{proton}\;\mathrm{therapy},},\mathrm{each}\;\mathrm{dose}\;\mathrm{achieving}\;\mathrm{the}\;\mathrm{same}\;\mathrm{specified}\;\mathrm{bioeffect}.$$

The actual prescribed dose of protons (often called the Cobalt Equivalent Gy or RBE-Gy, and more recently formalised as Gy (RBE), given to the patient is the intended photon dose divided by the operative RBE (which at the present time is assumed to be 1.1). It follows that an incorrect RBE will lead to an incorrect physical dose.

The relationship between LET and RBE is generally linear with RBE increasing up to a maximum value after which RBE falls, probably due to wasted local dose in a very small region that does not contribute to enhanced cell killing. In proton therapy the Bragg peaks are either ‘spread out’ by the passive scattering delivery method or are overlapped by the scanning technique, so that in each case the tissues receive a mixture of Bragg peak (high LET) and non-Bragg peak (low LET) regions, so that the average LET is around 1–2 keVmm^−1^, in the tumour region, although can be higher and up to 8–10 keV.mm^−1^ depending on the field arrangements and technique used [3]. The spreading out is non-uniform with respect to LET, such that the distal end region of the spread out Bragg-peak (SOBP) contains a greater proportion of peaks (and a higher LET) than the proximal region.

Now the average LET of conventional megavoltage radiotherapy is around 0.2 keV.mm^−1^ [4, 5], which has an immediate implication that the mid SOBP LET is around 6–9 times higher, with even higher values towards its end. Another complicating factor is that much of the research on LET and RBE (for protons and other forms of radiation) used low voltage X-ray beams, whose LET was already around 1–1.5 keV.mm^−1^, and which if used to estimated proton RBEs would underestimate the RBE by 5–15% or more depending on the experimental systems and energies used. Past conversions between low-voltage and high-voltage photons used fixed percentages that may change with different biological endpoints and at different doses.

For further understanding, it is necessary to describe the simple, but elegant mathematical relationship between radiation dose (*d*) and effectiveness (*E*) described by the linear-quadratic (LQ) model where:

*E = ad + βd*^2^, (1),

where *α* and *β* are radio-sensitivity (or cell killing) coefficients, with α predominating at low dose and β at high doses.

Now, for the same bioeffect denoted by E, protons and conventional megavoltage X-rays (photons) can be represented as follows:

E = proton effect = X-ray(photon) effect, or

**Figure 1.**
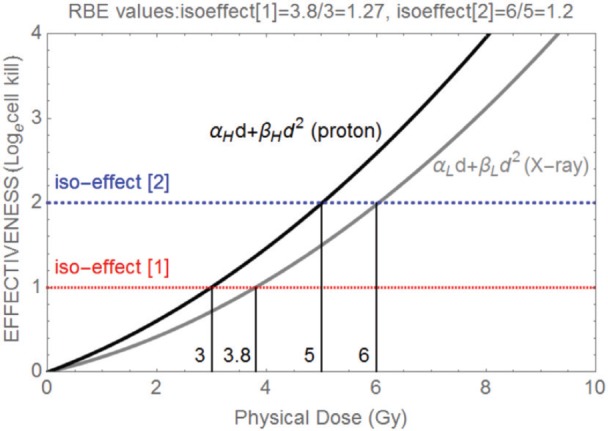
Demonstration of the RBE principle for two dose effectiveness curves produced by X-rays (photons) and protons (assuming α_L_ = 0.15 Gy^-1^, α_H_ = 0.24 Gy^-1^, β_L_ = 0.03 Gy^-2^, β_H_ = 0.032 Gy^-2^) and where two different iso-effect levels [1] and [2] are considered and their corresponding RBEs are shown. The numbers 3, 3.8, 5 and 6 refer to the physical doses where each iso-effect line meets each curve and so are respectively d_H_ followed by d_L_ for isoeffect [1] and d_H_ and d_L_ for isoeffect [2], so the RBE is given by d_L_/d_H_ in each case, as shown above the figure.

*E = α*_H_*d*_H_ *+ β*_H_*d*_H_^2^ *= α*_L_*d*_L_ *+ β*_L_*d*_L_^2^

where subscripts *L* and *H* refer to the low and high linear ionisation or LET states of each class of radiation. Note that two different doses are required for each class of radiation in order to maintain the iso-effect. The RBE is then the dose ratio *d*_L_/*d*_H_, which should always exceed 1. These relationships can be seen in Figure 1, where it can also be noted that the RBE is larger at lower effectiveness levels (this is due to the linear quadratic shapes of each curve).

In Figure 1, demonstration of the RBE principle for two dose effectiveness curves produced by x-rays (photons) and protons (assuming α_L_ = 0.15 Gy^−1^, α_H_ = 0.24 Gy^−1^, β_L_ = 0.03 Gy^−2^, β_H_ = 0.032 Gy^−2^) and where two different isoeffect levels [1] and [2] are considered and their corresponding RBEs are shown. The numbers 3, 3.8, 5, and 6 refer to the physical doses where each iso-effect line meets each curve and so are, respectively, *d*_H_ followed by *d*_L_ for isoeffect [1] and *d*_H_ and *d*_L_ for isoeffect [2], so the RBE is given by *d*_L_/*d*_H_ in each case, as shown above the figure.

The ratio *α/β* (expressed in units of Gy as in the case of dose), is a useful parameter that reflects the overall radiosensitivity and DNA repair capacity of a cell or tissue. It has many clinical applications, especially the biological effective dose (BED) concept [6] which allows iso-effective dose calculations, comparisons of different radiation schedules, dose rates and techniques.

Some general trends have been confirmed from LET-RBE experimental studies that have used a variety of radiation modalities, including fast neutrons, X-rays (of low energies), alpha particles, carbon, protons, and other light ions. These include the following:

1. Increasing LET elevates the *α* parameter more than the *β* parameter, both increments contributing to an increase in RBE [7].
2. The RBE is inversely related to the given dose [8–11], which follows logically from the finding that *α* increases more than *β.*
3. The maximum possible values of RBE are found (in the above references) with lower doses and depend on the cellular or tissue radio-sensitivity and DNA repair capacity: systems which are highly radio-sensitive have low RBEs and those which are radio-resistant have high RBE values. This is a direct consequence of the numerator dose (of the control radiation) in the RBE definition changing by more than the denominator dose (of the particle radiation) when dose per fraction changes, as in Figure 1.

Much of the earlier radiobiological research was done using fast neutrons, which remain relevant to proton therapy since most neutron beam ionisations are produced by intermediary recoil protons. Although neutrons do not exhibit the Bragg peak effect but are attenuated through tissue rather like an energetic X-ray beam, their radiobiological properties reflect the LET conditions within the Bragg peaks, where the RBE values can be broadly similar in the 2–3 range [12,13], although much higher than in the mid spread-out Bragg peak regions.

## Normal tissue margins (NTM) around a tumour

According to the ICRU definitions [5], the visible tumour volume is called the gross tumour volume (GTV); the further volume that includes a margin for microscopic tumour extension is called the clinical target volume (CTV); and a further margin that compensates for daily shifts in positioning of the patient relative to the beam, movements during treatment and penumbral beam effects is called the planning target volume (PTV). In practice, the CTV is determined by clinical risk factors and can include a margin of 0.5–3 cm in all or some directions around the tumour. The CTV to PTV margin is often around a further one cm, but the precise number will depend on the pathological features of the tumour itself.

In clinical practice, these volumes may include further variants due to the use of shrinking field techniques (commonly two or three CTVs), or in-field boost techniques, but the basic margins remain the same even if further volumes are defined at increasing distances in order to deliver lower normal tissue doses where lower risks of microscopic spread occur. Furthermore, after subtotal or ‘total’ surgical removal of a cancer, there may be little or no evidence of macroscopic tumour, but there is a necessity to cover a wide volume that will vary according to the extent of surgery performed and will consist entirely or almost entirely of normal tissue.

If the basic GTV, CTV and PTV are considered, it is important to realise that the volume of normal tissue treated beyond a tumour can exceed that of the tumour itself. For instance, consider a spherical 2 cm diameter tumour with an additional linear 1 cm CTV ‘margin’ beyond it (note that margins of up to 2 cm may be required for some histological tumour types).

The volume of normal tissue irradiated to the same dose as the tumour is then:

$$\frac{4}{3}\pi 1.5^{3}-\frac{4}{3}\pi .1^{3}=9.95\;{\mathrm{cm}}^{3},$$

but the tumour volume (the second term in the above equation) is only 4.19 cm^3^.

For larger tumours, there is inevitably a greater normal tissue volume, for example in the case of a 4 cm diameter tumour with a required 1 cm margin, then the volume of normal tissue included is:

$$\frac{4}{3}\pi 3^{3}-\frac{4}{3}\pi .2^{3}=\frac{4}{3}\pi (19)=78.59\;{\mathrm{cm}}^{3},$$

**Figure 2.**
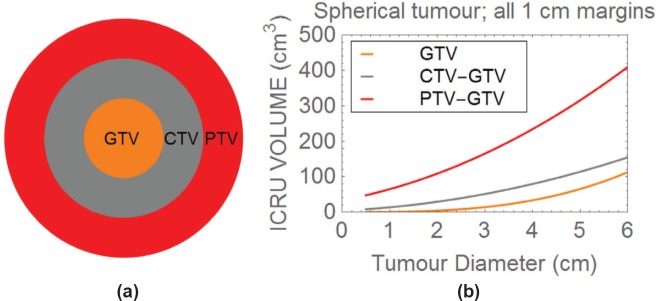
Plot of the three ICRU tumour volumes, with 1 cm margins between the GTV and CTV, and between the CTV and PTV.

This exceeds the tumour volume (again the second term of the last equation) which is only 33.51 cm^3^ by around 45 cm^3^. Further increments occur by adding a PTV margin, as shown in Figure 2.

Beyond the GTV, each volume is obtained by subtracting the neighbouring smaller volume, to show how much normal tissue is included between each volume.

In Figure 2(a), three concentric volumes are depicted representing the GTV, CTV and PTV. Figure 2(b) depicts how the ICRU volumes change with tumour diameter, assuming a spherical tumour and symmetrical 1 cm margins in all directions between each volume. Beyond the GTV, each volume is obtained by subtracting the neighbouring smaller volume, to show how much normal tissue is included in each of the CTV and PTV.

One can now appreciate that the normal tissue included in the full-dose region, which covers both the CTV and PTV can be critically important. Now if the normal tissue RBE in these regions exceeds that used in the prescription, overdosage of within these normal-tissue bearing volumes may occur. For organs at risk that are outside the PTV, then depending on the degree of dose sparing, normal tissue RBE`s would need to exceed the degree of sparing (in percentage terms) before causing toxicity. For example, if an important organ-at-risk (OAR) structure has a tolerance value of say 50 Gy in 25 fractions and receives 68% of the PTV dose of 72 Gy-RBE (where RBE is 1.1), then it is assumed that the OAR receives 48.96 Gy-RBE, which seems acceptable. If the relevant LET results in a dose-per-fraction adjusted RBE of say 1.2, then the actual equivalent dose will be (1.2/1.1) × 48.96 = 53.41 Gy, which exceeds tolerance and may cause a severe toxicity in a small number of patients (this could be blindness for example). The situation would be worse if say the RBE was to be 1.25, then the equivalent dose would be (1.25/1.1) × 48.96 = 55.63 Gy. Much higher RBE`s than 1.25 have been predicted for LET values around 8–10 keV.μm^−1^. Shrinking field and adaptive image guided techniques may sometimes be useful to reduce these normal tissue bearing volumes.

In many applications of radiotherapy, the gross tumour has been removed and treatment has to be given to a zone of risk determined by the propensity of tumour spread and the area of the excisional surgical procedure. The radiation treatment is therefore entirely given to normal tissue: there is no physical separation between tumour and normal tissue, which can contain separate volumes of risk. In such situations, proton therapy can only spare more distant normal tissues.

Consequently, great care is necessary. In some anatomical situations, the normal tissues within these margins are expendable as long as they do contribute significantly to the function of an organ system (as may be the case in the lung, where the volume of lung tissue rendered unable to perform gas exchange may be far less than the volume of lung removed after radical surgery which entails the minimum loss of an entire lobe). In other organs, such as liver, where there is also regenerative capability, some loss of normal tissue may be acceptable.

## Modelling the RBE

**Figure 3(a–c).**
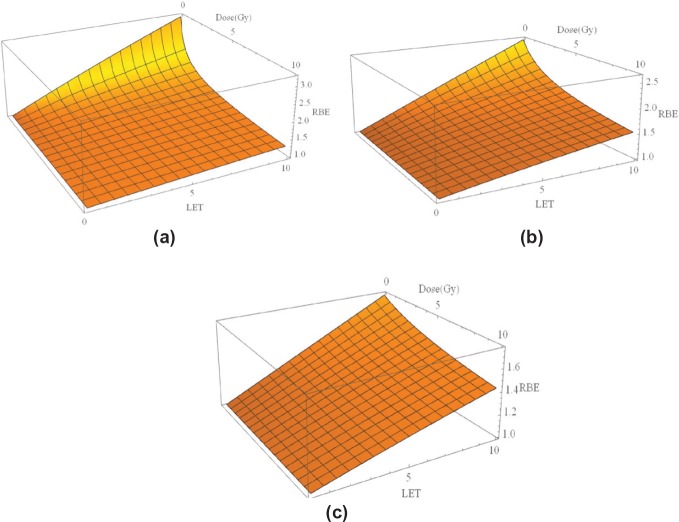
Three-dimensional plots of LET, dose per fraction and RBE for three different α/β values: (a): α/β = 2 Gy (representing highly fraction sensitive late reacting tissue such as spinal cord and brain). (b): α/β = 10 Gy (acute normal tissue effects and most rapidly growing moderately radio-sensitive tumours), and (c): α/β = 25 Gy (highly radiosensitive tumours).

There have been many attempts at modelling the increase in RBE with LET, using phenomenological or theoretical models or a combination of each approach. In terms of the linear quadratic model of radiation effect, they either relate the increased RBE inversely to the α/β ratio of the control radiation [14,15] or may for good reasons involve separate treatment of α [16], or α and β [17], coupled with saturation effects that limit the maximum possible increase but control the maximum possible RBE, which leads to a better overall description and relates well to the concept of both maximum and minimum limits to RBE at any value of LET, with the actual RBE being an intermediate value that depends on the given dose. This form of modelling, with separate treatment of α and β, is used to generate the graphical displays shown in Figure 3(a–c) where for three α/β values (2 Gy characteristic of the central nervous system, 10 Gy characteristic of many squamous cell carcinomas and 25 Gy characteristic of highly radiosensitive tumours such as lymphomas and many childhood tumours). The baseline LET value is set to 0.22 keVmm^−1^, being representative of megavoltage X-rays (photons) used in the clinic. The assumed control β values was 0.03 Gy^-2^ with α/β as specified. Further details can be found elsewhere [7,17].

**Figure 4 (a & b).**
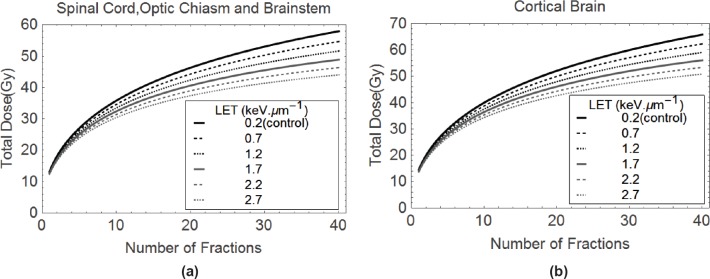
Plots of Total Dose and number of fractions isoeffective with (a) 50 Gy in 25 fractions, and (b) 60 Gy in 30 fractions, these doses being for the control megavoltage photon(X-ray) case at a LET of 0.2 keV.mm^−1^.

It is also possible to build up isoeffective plots for critical normal tissues such as the central nervous system. These are achieved by solving for the higher LET dose (*d*_H_) in the following iso-effect equation based on biological effective dose or BED [6,7,9,11,17], which is a transformation of the earlier equations achieved by collecting the radiosensitivity terms as α/β ratios:

LOW LET BED EFFECT = HIGH LET BED EFFECT,

which is symbolically represented as:

$$n_{L}d_{L}\left(1+\frac{\mathrm{dL}}{{\left(\frac{\alpha }{\beta }\right)}_{L}}\right)=n_{H}d_{H}\left({\mathrm{RBE}}_{\max}+\frac{{\mathrm{RBE}}_{\min}^{2}d_{H}}{{\left(\frac{\alpha }{\beta }\right)}_{L}}\right),$$

where *d*_L_ is the control dose per fraction, *n* are the number of treatments and RBE_max_ and RBE_min_ represent the limits of RBE at very low and very high dose, respectively, each given by *α*_H_*/α*_L_ and*√β*_H_*/√β*_L_. It can be noted that the same *α/β* ratio (that of the low LET) is used throughout, since the RBE limits are effectively multipliers of this basic ratio. In this way, a dose of 50 Gy in 25 fractions of 2 Gy, which denotes the conventionally used tolerance value for spinal cord tissue, provides 100 Gy (for α/β = 2) on the left-hand side. Providing the RBE limits are known with reasonable accuracy, then the equivalent dose for combinations of *d*_H_ and *n*_H_ can be found. Two graphical examples are presented in Figures 4 (a&b) for spinal cord and cortical brain radiation tolerances, where the isoeffective doses compensate for LET increases by a change in the RBE. In this way, clinicians can select reasonable dose-fractionation schedules that would protect critical normal tissues, or at least use these recommendations as boundary conditions not to be exceeded.

In order to provide an easier read out of RBE values than in 3-D plots, Table 1 estimations of RBE in nervous system tissues with α/β = 2 Gy, based on the radiobiological parameters shown and using the model in reference [7], which adjusts both α and β with LET, using 0.22 keV.mm^−1^ as the control LET.

The reduction of total dose required to maintain these iso-effects are shown for increasing values of LET that may be encountered in proton therapy

## Clinical outcomes

For many years, publications that provide outcomes (tumour control, survival, acute and late toxicities) after proton therapy have been relatively uncommon compared with technical reports of dose distributions and treatment delivery techniques [18], although the number of clinical publications is now increasing. Concerns have been expressed about children with very radiosensitive tumours, and where lower ‘photon equivalent’ doses are given – there remain unclear benefits in terms of tumour control, but no apparent increase in toxicity [19]. In such situations, the below tolerance doses used for most of the whole CNS tissue are below neurological tolerance, so that an RBE shift of say 8–10% will not necessarily produce more serious side effects [20]. The exception can be the relatively radio-resistant cerebellum and a small part of the more sensitive adjacent brainstem, where higher doses close to tolerance are given. There are reports of some cases of higher than expected brainstem toxicity, assessed by radiological changes, in ependymoma patients treated using a similar proton technique [21].

In one report [22], there was a significant serious neurological toxicity rate of 12.3%, a result that would not be tolerated with photon-based therapy, where local protocols are designed not to allow more than 1–2% risks, and often aim for below this range, while being capable of delivering high tumour doses using modern techniques. Also, the long-term results of children treated with protons in Japan show a significant rate of late complications: the grade 3 or higher late toxicities were 6%, 17%, and 17% at five, ten and twenty years respectively [23].

It is important that clinical groups publish their outcome data, and modify their protocols if necessary, as this will inform the cancer treatment community of what is presently being achieved and will increase overall confidence.

Another barrier appears to be that medical physicists often overemphasise the Bragg peak placement issues and minimise the radiobiology uncertainties, so that it becomes difficult to determine the cause of toxicity or failure to cure. Realistic assessments of both are needed, but it cannot be disputed that the range of known variation in RBE far exceeds that of Bragg peak positioning. This aspect must also be understood by the inevitable fact that displacement of Bragg peak position away from the ICRU target volumes might increase normal tissue toxicity outside the defined PTV but would be expected to be accompanied by reduced tumour control: there is no evidence that this has occurred, so it is reasonable to invoke RBE as being the main culprit.

## Discussion: how can radiobiology improve proton therapy?

The important influence of radiobiology in particle therapy should not be dismissed lightly, although some uncertainties exist in the accuracy of physical dose and LET distributions in treatment plans. It is difficult to separate clinical effects caused by uncertainties of dose placement (or dose intensity) and RBE. Shifting of dose away from target volumes could cause both enhanced toxicity and reduced tumour control, or either alone.

So many classical radiobiological facts show that RBE is a function of dose and the ionisation density (or linear energy transfer referred to as LET) and varies with cell type according to their intrinsic radio-sensitivity. It follows that RBE cannot be a constant value when taken in the context of the complexities of different tissues within the human body, the heterogeneity of cancer types, and the wide variety of doses used as well as the degree to which Bragg peaks are shifted or mixed with lower LET regions. The 1.1 RBE value used in the mid SOBPs has been extensively criticised [24] on the basis of the LQ theory, the use of short-term in vitro and in vivo assays that may not be appropriate to predict late tissue effects in the human, the choice of dose, the surprising inclusion of so many experiments where the control irradiation used relatively low-voltage X-rays that have their own 1.1 RBE effect, and a simplistic linear approach to data fitting. Furthermore, testing of some ‘variable’ RBE models in treatment plans is also gaining ground [25] and shows the potential for adverse clinical effects, although these may not show the full impact at higher doses per fraction.

When will the constant RBE be replaced? Will this be left to individual institutions to decide, or will some national and international bodies such as ICRU and ICRP intervene with improved guidelines? The decision-making process will depend on rational application of scientific (theoretical and experimental) and clinical data. This will ultimately depend on senior clinicians, not many of whom are experienced in more esoteric areas of radiobiology, which can also be said for many clinical physicists. The classical experimental radiobiologists, who have been aware of the current dilemmas, have not been in a sufficiently strong position to influence clinical practice in proton therapy.

Over the past 20 years, the present author was of the opinion that proton therapy would be of significant overall benefit but would need considerable further research including detailed RBE predictive studies to achieve optimised results [26–29], with the most appropriate funding model a mix of governmental research council and cancer charity contributions for more fundamental pre-clinical research portfolio and extending into the clinical phase. Many countries, including the United Kingdom, have instead adopted a health service based financial model to apply proton therapy, accepting that it is a therapy that has been shown to be of considerable benefit rather than being an experimental therapy. It would be unproductive if implementation in many countries will provide disappointing clinical outcomes, especially enhanced severe side effects arising from LET-RBE effects in the region close to the tumour. Reduction in patient referrals and funding could then lead to unfortunate closure of at least some proton centres. Better by far would be to implement rational strategies, perhaps within randomised clinical trials, to improve outcomes. Future decision makers for such policies will need to be suitably qualified and well-informed about the science and clinical base in order to even tentatively break away from the current conventional approaches. The present reports of higher than expected adverse outcomes should also stimulate this process.

A major challenge faces every proton therapy clinician: should they adopt different RBE values to limit toxicity and ensure better effectiveness in some situations? Should randomised control studies be done to test RBE allocations? For example, by allowing randomisation of patients to either the standard RBE or to an RBE of 1.2 in the normal tissues exposed to LET of 1–2 keV.mm^−1^. To compensate for this, a 1.1/1.2, or around 8% reduction in dose, with perhaps higher reductions required for larger LETs if found during the treatment planning process.

No physician would agree to deliberately under-dose paediatric tumours, but this may sometimes be the case if the tumour RBE is substantially less than 1.1, as predicted for the special situation of radiosensitive tumours (medulloblastoma, ependymoma, lymphoma and other solid radiosensitive children`s tumours). Physicians should note that the 1.1 RBE involves a dose reduction of 1/1.1, a reduction of around 9%. This important issue can be tested by using an RBE reduction of only 1.03, or not using an RBE at all (so that no risk is taken by reducing the tumour dose), provided that care is taken not to overdose critical structures where and RBE of 1.2 or above might be applied. Again this strategy could be tested within a randomised control trial [30]. Undoubtedly, this would imply tougher normal tissue constraints and demands on the physical beam arrangements in treatment planning.

Another major issue is what to do about very high LET`s at the distal end of each single treatment ‘field’ or volume, where LET`s could be 10 keV.mm^−1^ or more and high RBE`s are predicted. If these areas are close to critical normal tissues then dose reduction should be considered by reducing the number of scanned beams allowed in that position or by changing the dose profile of passively scattered beams accordingly. Further advances in treatment delivery can be expected and LET dose painting of tumours is being discussed and is expected to be implemented in clinical trials soon [31].

As the clinical practice of proton therapy evolves, it will be important to link this to an improved understanding of the radiobiological issues which impact on tumour and normal tissue responses.

## Declarations

None.

## Figures and Tables

**Table 1.** Estimated RBE values using the model described in references 7 and 17 but using a reference LET of 0.22 keV.mm−1.

Dose (Gy)	α/β = 2 Gy: Central Nervous System late effects with mean α = 0.07 Gy^−1^ (SD = 0.02 ).									
	LET = 1	LET = 1.25	LET = 1.5	LET = 1.75	LET = 2.0	LET = 2.5	LET = 3	LET = 4	LET = 8	LET = 10
d = 1.5	1.09	1.11	1.14	1.17	1.19	1.24	1.29	1.38	1.72	1.88
d = 1.8	1.08	1.10	1.13	1.15	1.17	1.22	1.26	1.35	1.66	1.80
d = 2	1.07	1.10	1.12	1.14	1.16	1.20	1.25	1.33	1.62	1.75
